# Biodiversity and Health in the Urban Environment

**DOI:** 10.1007/s40572-021-00313-9

**Published:** 2021-05-12

**Authors:** Melissa R. Marselle, Sarah J. Lindley, Penny A. Cook, Aletta Bonn

**Affiliations:** 1 University of Surrey, School of Psychology, Guildford, GU2 7XH UK; 2 Department of Geography, School of Environment, Education and Development, University of Manchester, Oxford Road, Manchester, M13 9PL UK; 3 School of Health and Society, University of Salford, Salford, M6 6PU UK; 4 Department of Ecosystem Services, Helmholtz Centre for Environmental Research - UFZ, Permoserstr. 15, 04318 Leipzig, Germany; 5 Institute of Biodiversity, Friedrich Schiller University Jena, Dornburger Straße 159, 07743 Jena, Germany; 6 Department of Ecosystem Services, German Centre for Integrative Biodiversity Research (iDiv) Halle-Jena-Leipzig, Puschstr. 4, 04103 Leipzig, Germany

**Keywords:** Biodiversity, Species richness, Public health, Anxiety, Disease, Nature-based solutions

## Abstract

**Purpose of review:**

Biodiversity underpins urban ecosystem functions that are essential for human health and well-being. Understanding how biodiversity relates to human health is a developing frontier for science, policy and practice. This article describes the beneficial, as well as harmful, aspects of biodiversity to human health in urban environments.

**Recent findings:**

Recent research shows that contact with biodiversity of natural environments within towns and cities can be both positive and negative to human physical, mental and social health and well-being. For example, while viruses or pollen can be seriously harmful to human health, biodiverse ecosystems can promote positive health and well-being. On balance, these influences are positive. As biodiversity is declining at an unprecedented rate, research suggests that its loss could threaten the quality of life of all humans.

**Summary:**

A key research gap is to understand—and evidence—the specific causal pathways through which biodiversity affects human health. A mechanistic understanding of pathways linking biodiversity to human health can facilitate the application of nature-based solutions in public health and influence policy. Research integration as well as cross-sector urban policy and planning development should harness opportunities to better identify linkages between biodiversity, climate and human health. Given its importance for human health, urban biodiversity conservation should be considered as public health investment.

## Introduction

Biodiversity underpins ecosystem functions that are essential for human health and well-being [1–3]. However, biodiversity is declining at an unprecedented rate [2], threatening the health and well-being of all humans. Growing urbanisation is an additional threat to both human health [4] and biodiversity [5]. Urban areas also experience increasing climatic pressures driven not only by global climate change developments but also by urbanisation and associated local urban heat island effects [6]. This is because cities contain larger amounts of surfaces with thermal and structural properties which enhance heat storage and inhibit its loss; fewer vegetated surfaces that contribute to cooling; and higher heat emissions due to human activity, such as traffic or air conditioning. As global urban cover is projected to increase to 1.9 million km^2^ with 5.2 billion people expected to live in urban areas by 2030 [5], action is needed to reduce future risks by designing healthy, liveable cities for both people and nature. Nature-based solutions may help to mitigate climate and health pressures in urban areas [7, 8•]. New alliances of the World Health Organization (WHO) and the Convention on Biological Diversity (CBD) are recognising this potential of aligning health, climate and biodiversity goals [9•], while concrete and actionable evidence is needed to operationalise health-promoting measures.

Of the large body of research on nature and human health [10–12], few studies examine the influence of ecological characteristics on health [13, 14•, 15]. Moreover, the extensive research on ecosystem service benefits to human health and well-being often lacks specifics on the biodiversity involved [1, 16]. Accordingly, we see a need to further develop knowledge of the ways in which specific elements of biodiversity itself matters for human health [17, 18••]. A first, simplistic approach to measuring nature, for example as the amount of greenspace, has enabled a surge of new research and can serve as an important indicator for urban health planning goals [15, 19]. Yet, it does not enable a clear understanding of how human health is influenced by the presence of, contact with, or change in different manifestations of biodiversity.

Research taking place over the past few decades has provided a much clearer picture of the connections between nature and human health in urban environments [11, 20, 21]. We now know much more about which elements of urban nature have positive and negative impacts and why those effects are seen. Emerging evidence underlines that benefits are not only due to the quantity of greenspace and bluespace in an area but also due to their quality. Biodiversity is a fundamental component of quality in this context, and therefore, research efforts are now turning to focus on understanding how and why biodiversity has an impact, and what this means for how we plan, design and manage urban areas of the future. This paper highlights the influences biodiversity of species and habitats has on human health and discusses new opportunities for understanding these relationships in order to conserve and enhance diversity for the health of both people and nature.

### Definitions of Biodiversity and Health

‘Biodiversity’ (Box 1) and ‘health’ (Box 2) mean different things to different people [2]. The definitions adopted here enable comprehension of the concepts of biodiversity and human health. Clear definitions can facilitate collaboration between the natural sciences, social sciences and health sciences in understanding biodiversity’s influence on human health.

Box 1 Definition of biodiversity

**Table:** 

*Biodiversity* is defined by the Convention on Biological Diversity (CBD) as “the variability among living organisms from all sources including, inter alia, terrestrial, marine and other aquatic ecosystems and the ecological complexes of which they are part; this includes diversity within species, between species and of ecosystems” [22]. Here, we use biodiversity in a broad sense to include the composition, configuration and diversity of specific species or habitats; the abundance and biomass of species; the functional traits of species (e.g. nutrient content, medicinal properties, colours, sounds, contagious properties); and the genetic composition (e.g. SARS-CoV-2) and identity of particular species (e.g. lion, robin, ticks, oak). In this article, the term ‘biodiversity’ will frequently be used in the text as shorthand for these elements of biodiversity. Importantly, biodiversity is more than just the amount of nature or greenspace. *Nature* as defined by Hartig et al. [11] refers to “physical features and processes of nonhuman origin that people ordinarily can perceive, including the “living nature” of flora and fauna, together with still and running water, qualities of air and weather, and the landscapes that comprise these and show the influence of geological processes”. The term greenspace is defined as outdoor areas dominated by vegetation (e.g. parks) or isolated green elements (e.g. street trees) [23]. These broad terms, which are often used in studies investigating the health benefits of natural environments, can limit the understanding of how variation in the ecological characteristics of the natural environment relates to health.	

Box 2 Definition of health

**Table:** 

*Health* is defined by the WHO [24] as “a state of complete physical, mental, and social well-being and not merely the absence of disease or infirmity”. Importantly, the WHO’s definition of health highlights factors that cause disease (pathogenesis), as well as those factors that promote health and well-being (salutogenesis). The definition of health includes three separate aspects of well-being. *Physical well-being* refers to the quality and performance of bodily functioning. This includes having the energy to live well, the capacity to sense the external environment and experiences of pain and comfort [25]. *Mental well-being* refers to the psychological, cognitive and emotional quality of a person’s life, which includes the thoughts and feelings that individuals have about the state of their life, and their experience of happiness (ibid). *Social well-being* concerns how well an individual is connected to others in their local and wider social community. This includes the number of social interactions a person has, the depth of their key relationships and the availability of social support (ibid).	

## Recent Research Findings

Biodiversity is a cornerstone of human health and well-being. This section details the current evidence on biodiversity’s influence on physical, mental and social health and well-being.

### Physical Health

There is a relatively large literature identifying indirect links between urban biodiversity and physical health. Understanding the connections between biodiversity and physical health is complicated by the use of multiple proxy indicators of biodiversity and health at a range of different scales [16, 26–28]. Studies using specific biodiversity and physiological indicators have the potential to cut through some of this complexity, but these are still relatively rare [29]. Despite the uncertainties, existing evidence is revealing a number of direct and indirect influencing mechanisms, and these can be usefully linked with one or more of the eleven interconnected bodily systems [30•].

One direct mechanism for the beneficial role of biodiversity on human health is associated with the ‘biodiversity hypothesis’ related to microbiome [31]. Symbiotic microbes within the human microbiome help explain healthy development of the immune system [31, 32] and healthy functioning of the digestive system [33]. The diversity of an individual’s microbiome is strongly related to their lifestyle, environment and exposure characteristics [31, 34]. People living in urban areas tend to have fewer opportunities to come into contact with beneficial microorganisms whether through dietary, airway or skin exposure pathways [31]. In one example, adolescent atopy was found to have statistically significant negative associations with the abundance and species richness of particular native flowering plants, with these plants being ~25% more abundant around the homes of healthy individuals [35]. In a cross-sectional observational study, primary school children exposed to higher fungal and fauna diversity around their schools were less likely to develop allergic sensitisation and to have improved lung function, respectively [36, 37]. Recent reviews have identified a lack of experimental and intervention studies with an explicit focus on testing the biodiversity hypothesis in the context of physical health [29, 30]. To address this gap, an urban biodiversity intervention in the gardens of a range of children’s day-care establishments in two cities in Finland has shown changes in both gut and skin microbiota and modified functioning of immune systems [34]. Other studies are now showing how interventions can also recolonise microbial communities lost through urbanisation [38]. Urban living can disrupt human microbiome biodiversity for any age group. For instance, pollution affects the richness of various environmental microbiome pools [34, 39]. Such disruptions can, in turn, lead to increased incidence of associated disease, such as in the digestive and urinary/renal bodily systems [26]. Urban dwellers are also more likely to experience dysbiosis, the negative cycle of continued human microbial imbalances [33]. Since microbiomes are partly inherited, dysbiosis can have persistent generational impacts especially in urban areas [31].

Biodiversity underpins a range of specific ecosystem functions known to contribute to human health [14•, 40], including mitigating the severity of urban environmental stressors [11, 18••, 21, 41]. Environmental noise is one such example. Here, stress-response mechanisms help explain how excessive noise impacts cardiovascular, respiratory, immune response and metabolic systems [42]. In turn, acoustic research shows how structural components of vegetation buffers noise, and demonstrates that dense and diverse planting schemes provide particularly effective noise barriers [43, 44]. Although urban populations may not always report strong satisfaction rates with environmental noise (reduction) in areas with greater land cover diversity [45], the physical processes through which vegetation attributes modify acoustics are nonetheless clear [43, 46]. People’s perceptions of the soundscapes of cities are thus important to consider, as the soundscape pleasantness of birds buffers the negative effects of traffic noise [47]. Air pollution is another example of an environmental stressor that biodiversity mitigates. Trees have been shown to reduce air pollution in cities, while they may also emit volatile organic compounds (VOCs), and the choice of right tree species matters to urban planning [48, 49]. Vegetation with higher structural complexity and density can also be an effective barrier to ultrafine particles from roads [50]. Nevertheless, the mitigating effect of urban vegetation on air pollution is still under debate. This is due to complex chemical and physical interaction of plants with the surrounding air depending on vegetation structure (e.g. planting density) and specific functional traits (e.g. leaf area, water use strategy, pollen production, VOC and ozone production) [48, 51–53]. Reducing exposure to extreme heat is the third example of how biodiversity mitigates environmental stressors. Extreme heat is of particular concern for the future due to climate change and more people living in urban areas, who may experience exacerbated urban heat island effects as well as higher vulnerabilities due to ageing populations [54–56]. Vegetation in cities can reduce these heat island effects by decreasing the air temperature through evapotranspiration and/or shading [57]. Vegetation abundance, structural characteristics, taxonomic diversity, species traits (e.g. leaf area, pigmentation and canopy structure), composition, functional diversity and functional identity are all known to affect the extent of cooling provided [14•, 30, 58–60].

In addition to mitigating urban environmental stressors, the biodiversity of urban ecosystems has been reported to have a net positive influence on a range of other beneficial functions and ecosystem services, too. They include pollination, soil protection and fertility, water quality regulation and pest control [14•, 58]. Health influences can occur irrespective of direct contact with biodiverse places, e.g. when flood attenuation or water quality regulation occurs through wetlands upstream of cities. However, the abundance and physical proximity of areas of biodiversity are undoubtedly important for attaining health and well-being benefits [61]. Indeed, the greater propensity for people to engage in physical activity if there are pleasant, open spaces near to home is also a major driver of health outcomes, due to both the greater likelihood of beneficial exposures and the physical health benefits of the exercise itself [62–64].

### Mental Health

Natural environments and greenspaces are beneficial for mental health and well-being [13, 15, 65]. While a large body of literature focuses on the spatial extent of a natural area or the amount of time spent in greenspace, fewer studies examine the influence of biodiversity in greenspaces on mental health and well-being [13, 15, 66]. The following discussion of the literature is organised by the tiers of biodiversity: ecosystems or habitats, species communities and single species [67].

A rich diversity of ecosystems and habitat types may improve mental well-being, but not mental health [68•]. Several studies show no significant relationship between biodiversity of ecosystems or habitats and mental health [69–72]. With regard to mental well-being, there is mixed evidence, and this relates also to spatial scale of investigations. Fuller et al. [73] could show a positive association of the number of habitat types with psychological well-being. Greenspaces with higher biodiversity were related to better well-being compared to greenspaces low in biodiversity [74]. An investigation of forest habitats, however, found forest habitats of intermediate biodiversity linked to greater positive reported emotions, than forests of high and low diversity [75]. More diverse ecosystems or habitats (assessed with the Shannon diversity index of land cover and land use) were positively associated with greater quality of life in Finland [72], but not with psychological well-being in England [76•]. In other studies, no effect was found for biodiversity on mental well-being for different ecosystems or habitats [77–79], protected areas [80] or different greenspace types [81].

There is some evidence that species richness of plants or animals can have a positive association on mental health and well-being [68•]. There is mixed evidence on the influence of bird species richness on mental health, with some studies showing a significant association at large spatial scales [82, 83] and in an experimental laboratory setting [84], and others at the neighborhood scale showing no relationship [85]. Plant species richness was associated with improved mental health at the regional district scale in Germany [83]. Although species richness of street trees at the very local scale (100 meters around the home) was not related to antidepressant prescriptions [86•]. With regard to mental well-being, greater tree and plant species richness was related to better mood [84, 87] and psychological well-being [73]. Species richness of flora and fauna was positively associated with subjective well-being [88]. Greater bird species richness was related to greater levels of life satisfaction [89, 90•], positive affect [84, 91] and psychological well-being [73, 76•] at several spatial scales. However, no relationship was found between mammal, megafauna and tree species richness on life satisfaction at large spatial scales [90•]. At the local scale, no relationship of butterfly species richness with psychological well-being could be ascertained [73, 76•], while a negative association was found between plant species richness and psychological well-being [76•].

When information on the level of species richness in an urban area is not available, a proxy measure may be used [91]. This proxy measure is perceived biodiversity, i.e. a survey respondent’s rating of the number of different species that they think is present in an environment [92]. No study, to date, has examined the relationship between mental health and perceived species richness [68•]. Regarding mental well-being, positive associations were found between perceived species richness of animals and plants and mood [91, 93]. While relationships with scientifically recorded biodiversity were nonsignificant or even negative, associations were found to be positive for perceived species richness of birds, butterflies and trees and psychological well-being [76•]. Marselle et al. [94, 95] found no associations between perceived species richness of plants. Perceived plant species richness was positively related with restorative affect [96], but not subjective well-being [97]. No influence was found for the perceived number of native species or the perceived number of native insects on restorative affect [96]; this may be because perceived biodiversity depends on the visibility of the species and the extent to which is perceived as different [98].

Abundance of specific distinctive taxonomic groups may be important for mental health and well-being, as they may be more noticeable than species richness [76•]. Indeed, studies that found nonsignificant results with species richness found significant results with abundance—and vice versa at different spatial scales. Regarding mental health, Cox et al. [85] found higher bird abundances in the afternoon—but not species richness—were associated with less depression, anxiety and stress in England. Marselle et al. [86•] found that abundance of street trees, but not species richness, significantly reduced the risk of antidepressant prescriptions for individuals with low socio-economic status in the city of Leipzig. In contrast, Methorst et al. [83] found no association between bird abundance on mental health but found a significant association with bird species richness. Regarding mental well-being, people viewing photographs with greater abundance of fish/crustaceans reported greater reported happiness than when viewing photographs with lower abundance—although no effect was found with species richness [99]. These four studies highlight the importance of measuring species richness and abundance together, in order to discern the independent contribution of species richness [98].

Importantly, spatial scale may be a very important factor, as different causal mechanisms may account for the influence of the different tiers of biodiversity on mental health and well-being. For example, the mechanism driving the influence of species richness at the regional district scale may be related to overall landscape qualities that may promote mental well-being, while species richness at the local scale might be more related to stress reduction or attention restoration that promotes mental health and well-being [18, 68, 83, 116]. Indeed, Marselle et al. [94, 95] did not find a direct relationship between perceived bird species richness and mood, but found an indirect relationship mediated by perceived restorativeness [95]. Further research is needed to unravel these mechanisms.

### Social Health

Social well-being is an important aspect of health. While the influence of greenspace on social interactions and social cohesion in one’s neighbourhood has been extensively studied [100–104], fewer studies have explored the association between biodiversity and social dimensions of health [68, 105•]. Engaging in nature conservation volunteering can facilitate social interaction [106]. Biodiverse environments, such as in neighbourhoods with more trees, may provide a setting for social interaction with others, which is likely to increase social cohesion [101, 107]. No relationship, however, was found between the vegetation complexity of a visited greenspace and social cohesion [108]. In contrast, neighbourhood well-being, a measure of self-reported satisfaction living with one’s neighbourhood environment, was positively associated with the species richness and abundance of birds as well as vegetation cover [89, 109].

Given that contact with nature can increase social interactions, social cohesion and prosocial behaviour [110, 111], future work should examine the relationship between social well-being and the biodiversity of different types of natural environments. Such insights into the social impact of biodiversity could contribute to efforts to facilitate pro-environmental behaviour change [110], contribute to sustainability [112] and mitigate the potential negative impacts of increased urbanisation.

## Outlook

### Future Research Directions

This brief review demonstrates some of the evidence that biodiversity contributes to better health and well-being in urban areas. This is important because of the increasingly urbanised character of human populations and the greater health and well-being stresses which come with urban living, such as air pollution and extreme temperatures, despite the greater proximity of health services. While several reviews already reported direct relationships between biodiversity and human health outcomes [30, 68, 105, 113•, 114, 115•], they do not shed light on the causal pathways through which biodiversity may work to establish those relationships. This lack of mechanistic understanding of pathways linking biodiversity to human health limits the application of nature-based solutions in public health [26]. In order to facilitate cross-sector integration between biodiversity conservation and public health [9], it is necessary to better identify and characterize the causal pathways linking biodiversity to human health.

Consequently, a key research challenge is to investigate the causal pathways linking specific elements of biodiversity to human health [17, 105, 113•] and test theoretical frameworks [18••, 116•]. How exactly does biodiversity influence human physical, mental and social health? Four domains of pathways have been proposed by Marselle et al. [18••]: (1) reducing harm (e.g. provision of medicines, decreasing exposure to air and noise pollution), (2) restoring capacities (e.g. attention restoration, stress reduction), (3) building capacities (e.g. promoting physical activity, transcendent experiences) and (4) causing harm (e.g. dangerous wildlife, zoonotic diseases, allergens). These four domains highlight the effects—both beneficial and harmful—that biodiversity can have on human health. Future research should empirically test this theoretical framework by investigating the causal mechanisms underlying biodiversity’s influence on human health.

Another key topic for biodiversity-health research is to understand which aspects of biodiversity matter and their perception. It is not clear to what extent perceived biodiversity measurements [91, 92] coincide with actual, scientifically recorded, biodiversity measurements. While some studies show a relationship of actual biodiversity with health and well-being measures [73, 91, 97, 117], other studies have found no relationship [76, 118]. When measures of actual and perceived do not coincide, this suggests that different things are being measured in the perceived biodiversity assessment, rather than actual biodiversity [98]. As such, perceived biodiversity measures cannot replace a measure of actual biodiversity [92] and vice versa. Future studies could usefully investigate whether perceived biodiversity mediates the effect of actual biodiversity on human health [68•].

A third key research frontier is the exploration of contact with biodiversity, which is defined as both exposure to and experience of biodiversity [18••]. Exposure is defined as the amount of contact that an individual or population has with biodiversity [18••, 20], and can be measured in two ways. The first is actual exposure to biodiversity, based on the frequency (how often) and duration (how long) a person or population has had contact with biodiversity [108, 119]. The second is a proxy measure of actual exposure, where the proximity or availability of biodiverse habitats, species communities or specific species within a geographical area near to a person’s location (e.g. neighbourhood, state, country) is used as an indicator for exposure [19, 20]. But, the vicinity of biodiversity and potential accessibility does not always equate to actual accessibility and actual exposure, i.e. that a person will come in contact with biodiversity. Approaches to exposure measurement do not capture the experiential aspects of contact with biodiversity—the experience of biodiversity. As people may experience biodiversity differently [74, 120], one’s experience of contact with biodiversity may be highly relevant for individual health effects [10]. For example, humans experience biodiversity through the five senses, which could account for differential impacts on health outcomes [121]. There are four types of biodiversity experiences based on whether we are exposed to the specific elements of biodiversity directly (e.g. in a park) or indirectly (e.g. through a window) and whether we interact with biodiversity intentionally or incidentally as a by-product of another activity [18••]. The type of experiences people have with biodiversity may influence health outcomes and mediating pathways [18••]. Future studies need to investigate actual exposure to biodiverse environments or species in order to unravel ‘dose-response’ relationships [122]. Investigating how the senses in which we can experience biodiversity and the specific types of experiences with biodiversity elements affect health outcomes is an urgent research need for understanding biodiversity-health relationships [18••, 20].

### Biodiversity Loss Is a Public Health Issue

Biodiversity is essential for the survival of the human species. Therefore, those whose role is to protect the public’s health should join the campaign against climate change and other threats to biodiversity [123]. At the global and government levels, cross-sector efforts should ensure that biodiversity, climate and health considerations are jointly integrated into government-wide and worldwide strategies. A good example of this is the UK environment strategy, which incorporates human health as a central goal [124]. Likewise, health strategies should also emphasise the importance of a healthy natural environment.

The biodiverse natural environment should be considered as a foundation for public health. Modern public health must also address health inequalities, whereby low socio-economic status can lead to up to 10 years life lost [125]. However, there is also inequality in access to natural environments, with more socially and economically deprived people living more likely in urban areas that are denuded of biodiversity [126], confounding efforts to establish the relationship between access to biodiverse environments and health. In analyses that control for social deprivation, health outcomes are shown to be related to several characteristics of greenspace (e.g. [61]). Although it is not possible to prove causality, the precautionary principle would suggest that investment in creating and maintaining natural environments in socially deprived areas will benefit public health and reduce health inequalities.

A key role of a public health professional is as a commissioner for health interventions. Such ‘green interventions’ should support lifestyles that benefit both humans and the environment, such as investments in cycling and walking infrastructure. Some of these ‘green interventions’, such as volunteering conservation projects, can have the dual impact of enhancing natural environments while simultaneously bringing human health benefits [127•]. Such health interventions are based on the concept of ‘salutogenesis’ whereby the emphasis is on supporting well-being before it becomes necessary to treat disease [128]. ‘Social prescribing’ connects people to holistic interventions, and green interventions fit very well within this salutogenesis model [127•].

However, to promote green health interventions with funding and embedding in strategies requires evidence of their effectiveness. As these interventions are complex and difficult to evaluate, those who fund research and interventions must understand and embrace this complexity. Research that quantifies the human health benefits and cost savings from investing in public greenspaces is needed to build persuasive evidence for investment. To foster the evidence base, cost-benefit analyses can assess the effectiveness of nature-based solutions on human health outcomes, such as reducing health care costs for noncommunicable diseases like depression [20, 86•], nature conservation and climate change mitigation. Scenario building and statistical modelling can assist in forecasting the effects of further biodiversity losses or gains for human health and thereby inform management priorities. This will help foster informed decision making as well as appropriate indicator development to monitor biodiversity and health trends to adapt integrated management and policy accordingly.

### Policy Support for Biodiversity and Health

The increasing relevance of biodiversity for health and well-being is increasingly recognised in global and regional policy development [9, 129, 130], such as the UK Government report on the economics of biodiversity [131], the Intergovernmental Science Policy Platform on Biodiversity and Ecosystem Services (IPBES) Global Assessment [2] or the IPBES pandemic report [132]. Since 2012, the WHO has collaborated with the CBD to raise awareness of the interconnections between biodiversity and human health [9•, 115]. While progress is being made linking the biodiversity and public health sectors [133], ‘silo-thinking’ is still common, and cross-sectoral approaches still need to be embraced by policy [123]. The current COVID-19 pandemic has highlighted the severe health consequences of unsustainable biodiversity management, as well as the importance of urban greenspaces for fostering physical and mental health and well-being and reducing health inequalities [134, 135]. The PBES has recently published a workshop report on biodiversity and pandemics [132] and integrates wider human health considerations in their current IPBES Nexus Assessment on the interlinkages among biodiversity, water, food and human health (https://ipbes.net/nexus).

Biodiversity-related public health threats as well as opportunities can only be addressed by integrating public health and environmental perspectives [127•]. To implement actions, policy frameworks are needed to assure that human health is included as integral to biodiversity conservation policies [9•]. Likewise, biodiversity should be considered in public health policies as well as spatial and urban planning [136]. The international policy discussions on the post-2020 global biodiversity framework of the CBD, the European Biodiversity Strategy and the European Green Deal [137] provide pertinent leverage points to strengthen the biodiversity-health policy agenda.

### Designing Cities for Both People and Nature

Urban planning decisions, such as fostering nature-based solutions in cities, can form proactive solutions to tackle both human health challenges and biodiversity loss while mitigating and adapting to climate change [7, 20, 138]. While urban planning policies explicitly consider nature conservation and climate change, the impacts of city planning decisions on human health is often considered implicitly [136]. Addressing health issues in urban planning is necessary for the creation of sustainable healthy cities that are able to cope with future developments, such as rising rates of mental health and cardiovascular diseases, future pandemic lockdowns and increasing climate change–related impacts. For this reason, interdisciplinary cooperation between urban planning, biodiversity experts, climate modellers and the health sector should be strengthened.

## Conclusions

With this review, we provide an overview of the rapidly developing research field of biodiversity and human health linkages with a focus on urban areas. We discuss the importance of exposure and experience of biodiversity for attaining health effects and develop a research agenda to quantify health benefits and unravel the specific causal mechanisms through which biodiversity affects human health. This understanding will enable urban planners and policy makers to harness opportunities for linking biodiversity, climate and public health agendas and to invest into biodiversity as foundation of our health.
